# Absence of localization in disordered two-dimensional electron gas at weak magnetic field and strong spin-orbit coupling

**DOI:** 10.1038/srep33304

**Published:** 2016-09-15

**Authors:** Ying Su, C. Wang, Y. Avishai, Yigal Meir, X. R. Wang

**Affiliations:** 1 Physics Department, The Hong Kong University of Science and Technology, Clear Water Bay, Kowloon, Hong Kong; 2 HKUST Shenzhen Research Institute, Shenzhen 518057, China; 3 Department of Physics, Ben-Gurion University of the Negev, Beer-Sheva, Israel; 4 Department of Physics, NYU-Shanghai University, Shanghai, China

## Abstract

The one-parameter scaling theory of localization predicts that all states in a disordered two-dimensional system with broken time reversal symmetry are localized even in the presence of strong spin-orbit coupling. While at constant strong magnetic fields this paradigm fails (recall the quantum Hall effect), it is believed to hold at weak magnetic fields. Here we explore the nature of quantum states at weak magnetic field and strongly fluctuating spin-orbit coupling, employing highly accurate numerical procedure based on level spacing distribution and transfer matrix technique combined with one parameter finite-size scaling hypothesis. Remarkably, the metallic phase, (known to exist at zero magnetic field), persists also at finite (albeit weak) magnetic fields, and eventually crosses over into a critical phase, which has already been confirmed at high magnetic fields. A schematic phase diagram drawn in the energy-magnetic field plane elucidates the occurrence of localized, metallic and critical phases. In addition, it is shown that nearest-level statistics is determined solely by the symmetry parameter *β* and follows the Wigner surmise irrespective of whether states are metallic or critical.

The one-parameter scaling theory (1PST) of localization^1^,^2^,^3^,^4^ has been instrumental in our current understanding of the metal-insulator transition (MIT) in disordered non-interacting systems. This theory assumes that the scaling function *β*(*g*), determining how the dimensionless conductance *g* changes with system size, depends only on *g* itself, and predicts that the occurrence of a MIT depends on the system dimensionality and its symmetry under time reversal (TR) and spin rotation (SR)^5^,^6^,^7^,^8^,^9^. In two dimensions (2D), for both the Gaussian orthogonal ensemble (GOE), where TR and SR symmetries are preserved, and the Gaussian unitary ensemble (GUE), where TR symmetry is violated, the 1PST asserts that all states are localized. On the other hand, for the Gaussian symplectic ensemble (GSE), where TR symmetry is preserved while SR symmetry is violated, there is a MIT. Thus, according to 1PST, despite the presence of spin-orbit scattering (SOS), even an infinitesimal magnetic field that breaks TR causes all states to be localized. At high magnetic fields, the occurrence of the quantum Hall effect indicates that extended states do exist, since in this regime, 1PST should be modified to incorporate two scaling parameters (e.g. the longitudinal conductance and the Hall conductance)^10^,^11^,^12^,^13^. The question addressed in this work is whether 1PST is still valid (as is widely believed) at weak magnetic fields and spatially fluctuating SOS. Our answer is negative. We show that under these conditions, the band of extended states that exists at zero magnetic field persists at weak magnetic fields, and eventually, with increasing magnetic field, crosses over at some critical field *B*_*c*_ into a band of critical states that has been shown to exist at strong magnetic fields^14^. For 0 ≤ *B* < *B*_*c*_ the bandwidth [−*E*_*c*_(*B*), *E*_*c*_(*B*)] between the two mobility edges is a slowly decreasing function of *B*.

To substantiate our claim, we study the nature of non-interacting electronic states in 2D under the influence of weak magnetic field, disorder potential and strongly fluctuating SOS, and carry out two kinds of numerical calculations: The first one studies the nearest level spacing distribution in various energy regimes, in order to identify the localized phase and the appropriate universality classes^5^,^6^,^7^,^8^,^9^. The second one consists of highly accurate procedure for identifying MIT, based on the transfer matrix technique and finite-size scaling arguments.

## Results

### Model

In weak magnetic fields, the Landau levels mix and projection on the lowest Landau level is meaningless. An appropriate and convenient procedure is then to consider a tight-binding model for 2D electrons hopping on a square lattice of unit lattice constant. The lattice sites are labeled as *i* = (*n*_*i*_, *m*_*i*_), with 1 ≤ *n*_*i*_ ≤ *L* and 1 ≤ *m*_*i*_ ≤ *M* integers. The Hamiltonian reads,


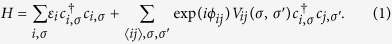


Here 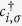 (*c*_*i*,*σ*_) is the electron creation (annihilation) operator at site *i* with spin projection *σ* = ±, and 〈*ij*〉 denotes nearest-neighbor lattice sites. The on-site energies *ε*_*i*_ are randomly distributed in [−*W*/2, *W*/2], (hereafter we take *W* = 1 and as long as 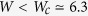, the results are similar. The model does not support any extended states for *W* > *W*_*c*_.), and the magnetic field is introduced by the Peierls substitution in which phase factors 
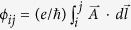
 multiply the hopping amplitudes, where 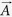 is the vector potential^15^,^16^. The dimensionless parameter *B* is defined such that magnetic flux through a unit cell is *Bϕ*_0_ where *ϕ*_0_ ≡ *hc*/*e* is the quantum flux unit. Accordingly, *B* is a measure of the magnetic field strength in this lattice model. The SOS is encoded by random *SU*(2) matrices *V*_*ij*_ acting on the electron spin that hops between sites *i* and *j*, defined as,


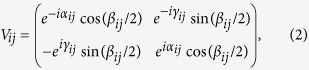


where *α*_*ij*_ and *γ*_*ij*_ are uniformly and independently distributed in a range [0, 2*π*], while cos*β*_*ij*_ is uniformly distributed in [−1, 1]. This model is hereafter referred to as the 2DSU model. For *B* = 0 it displays the (so called) symplectic MIT, pertaining to systems with conserved TR and broken SR symmetries, as also predicted within 1PST^17^. For strong magnetic field (e.g. *B* ≥ 1/5) the 2DSU model exhibits a Berezinskii-Kosterlitz-Thouless transition (BKTT) between a band of localized states and a band of critical states^14^. In the following we concentrate on the physics at weak magnetic fields, (explicitly, we even consider *B* < 10^−4^).

### Level statistics

Consider first the distribution *P*(*s*) of nearest level spacings *s* (in units of the mean level spacing). This analysis enables the distinction between localized and extended states, and in the latter case, identification of the relevant universality class: More concretely, for localized states, it is expected to follow the Poisson distribution *P*_Loc_(*s*) = exp[−*s*], while for extended states, *P*_*β*_(*s*) is specified by the symmetry parameter *β* = 1, 2, 4 (corresponding respectively to GOE, GUE and GSE). These three distributions are excellently approximated by the Wigner surmise expressions *P*_*β*_(*s*) = *C*_1_(*β*)*s*^*β*^exp[−*C*_2_(*β*)*s*^2^]. (The constants *C*_1_ and *C*_2_ are determined by normalization conditions for probability and unit mean level-spacing 〈1〉 = 〈*s*〉 = 1).

**Figure 1:**
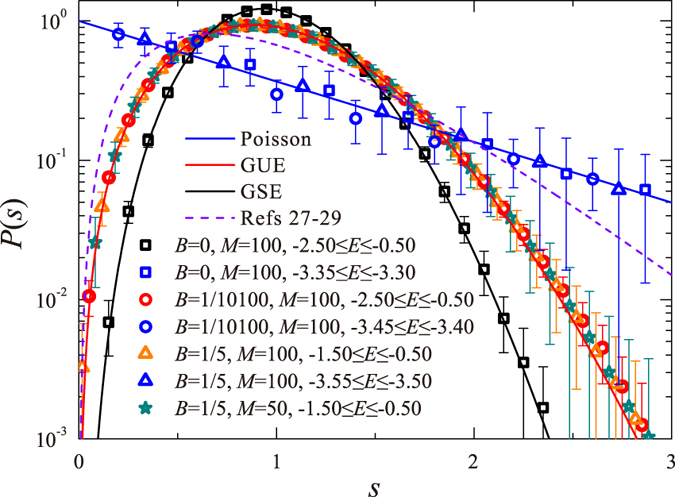
*P*(*s*) for *W* = 1, various magnetic field strengths *B* = 0, 1/10100, 1/5, various system sizes *M* = 50, 100, and in various energy ranges. Data are averaged over 1500 ensembles. It is evident that for *B* > 0 (no matter how small), *P*(*s*) corresponding to both critical and extended states fits well into the Wigner surmise for GUE (red solid line), whereas *P*(*s*) corresponding to extended states at *B* = 0 agree with the Wigner surmise for GSE (black solid line). The dashed line corresponds to the distribution suggested in refs 27, 28, 29 assuming *ν* = ∞ ⇒ *γ* = 1 since the localization length at a BKTT diverges faster than a power-law. For localized states with energies −3.55 ≤ *E* ≤ −3.50 far from BKTT mobility edge (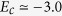 for *B* = 1/5), whose localization length is much smaller than the sample size (*M* = 100), *P*(*s*) agrees with the Poisson distribution (blue solid line).

For the actual computation, a finite lattice of size *M* × (*M* + 1) is considered and periodic boundary conditions are imposed on both directions using the almost antisymmetric gauge (see methods). That makes it possible to treat a weak field 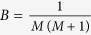. The Hamiltonian (1) is diagonalized, yielding all eigenvalues {*E*} and normalized wave functions {*ψ*_*E*_(*n*_*i*_, *m*_*i*_)} for each value of *B* and *M*. As shown in Fig. 1, *P*(*s*) for B = 0 and strong SOS displays, for a wide energy range −2.5 ≤ *E* ≤ −0.5, GSE statistics (data in black squares, theory in black curve). It suggests the existence of a band of extended states within the symplectic ensemble, commensurate with the prediction of 1PST^17^. Remarkably, adding a single flux through the entire area, corresponding to *B* = 1/10100 for *M* = 100 (red circles) is already sufficient to modify *P*_*β*=4_(*s*) into *P*_*β*=2_(*s*), where the level statistics follows the GUE Wigner surmise (red line in Fig. 1). In any case, the fact that in both cases *P*(*s*) follows the Wigner surmise and not Poisson distribution indicates that these are metallic-like states, where level repulsion occurs at small *s*. This behavior persists for different system sizes and for all *B* > 0. On the other hand, for energies below the mobility edge (blue shapes and curve in Fig. 1), *P*(*s*) obeys Poisson statistics, as expected for localized states. Thus, our analysis of nearest level spacing distribution suggests that states in the same energy range (as for *B* = 0) are still extended at finite magnetic field even though this 2D system now belongs to the unitary class. The wide range of parameters and energies where the GUE statistics has been observed, substantiates that this result is robust, namely, it is not due to finite size effects.

### Localization length

**Figure 2:**
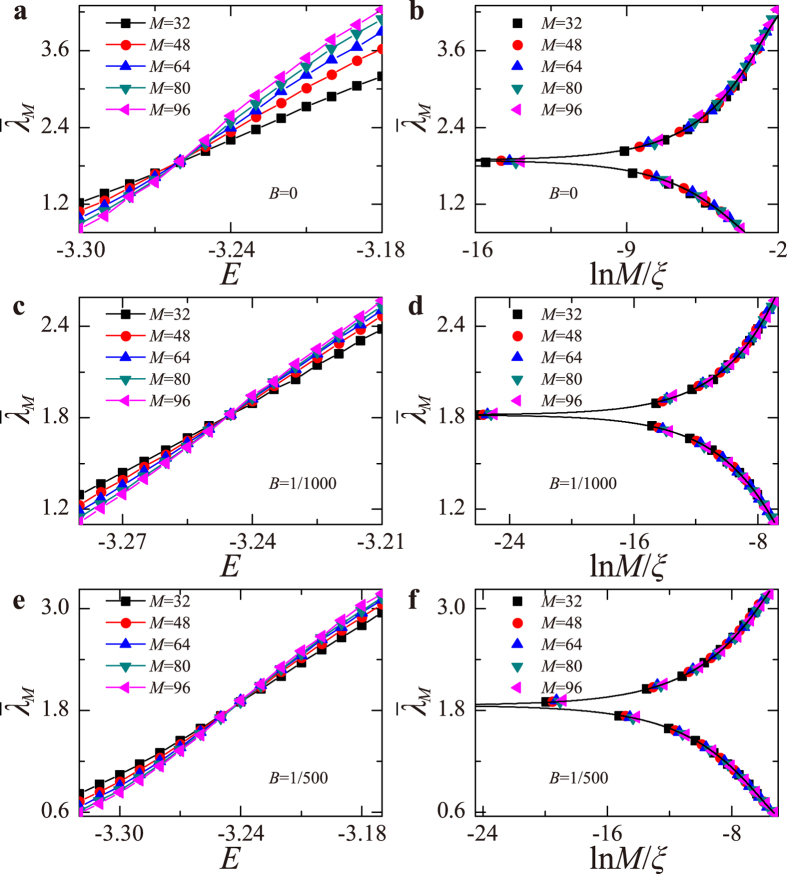
The left panel displays 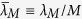 vs *E* calculated for disorder strength *W* = 1 and for three values of the magnetic field (**a**) *B* = 0, (**c**) *B* = 1/1000, and (**e**) *B* = 1/500 for *M* = 32 (squares), 48 (circles), 64 (up-triangles), 80 (down-triangles), and 96 (left-triangles). The scaling function obtained from (**a,c,e**) by collapsing data of 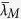 near the transition points into a single curve *ξ* ~ (*E* − *E*_*c*_)^−*ν*^ are shown in (**b**) for *B* = 0, (**d**) for *B* = 1/1000, and (**f**) for *B* = 1/500.

In order to corroborate our finding on the existence of extended states at weak magnetic field (that is so far based on level spacing analysis of finite size systems), we directly evaluate the localization length *ξ*(*E, B*) of the 2D system (up to a multiplicative constant) employing the transfer matrix technique^18^,^19^. Within this procedure, one evaluates the localization length *λ*_*M*_ of a stripe of width *M* and (virtually infinite) length *L* > 10^6^. According to the scaling analysis, the renormalized localization length of the strip, 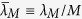, increases (decreases) with *M* for extended (localized) states and is independent of *M* for critical states. For the 2DSU model, Fig. 2(a,c,e) display 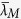 vs *E* for *B* = 0, *B* = 1/1000, and *B* = 1/500. It is clear from these figures that the system undergoes an Anderson MIT, since all curves for different *M* cross at two mobility edges at which 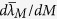 changes sign. The results of Fig. 2(a) just reconfirm the familiar symplectic MIT, but the MIT displayed in Fig. 2(c,e) occurring at mobility edges *E*_*c*_ = ±3.245 and ±3.242 is novel, and agrees with the conclusion based on level-spacing analysis: In the presence of strong SOS fluctuations, a band of extended states occurs in 2D systems even when its Hamiltonian breaks TR symmetry.

### One parameter finite-size scaling

To substantiate that these results are not merely due to finite size effects, we employ the one parameter finite-size scaling formalism, which is based on the hypothesis 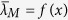, where *x* = *M*/*ξ* = *CM*/(*E* − *E*_*c*_)^−*ν*^. Here *C* is a constant and *ν* is the localization-length critical exponent. For optimal values of *E*_*c*_ and *ν*, the scaling function *f*(*x*) should be smooth (actually there are two functions, one for the insulator and one for the metallic side). The numerical values of *ν* characterize the universality class of the MIT^20^. In Fig. 2(b) the different curves of Fig. 2(a), when plotted as function of *x*, indeed collapse on a smooth curve that represents the scaling function *f*(*x*). Here, for *B* = 0, this result reconfirms the criticality of the symplectic MIT. The value of *ν* (see first row of the Table 1) agrees with previous ones^20^,^21^,^22^. Remarkably, inspection of Fig. 2(d,f) shows that the collapse scenario occurs also at finite magnetic field, namely the different curves in Fig. 2(c,e) fall on a single smooth curve. Moreover, for these novel MIT at *B* > 0, the dependence of *ν*(*B*) on *B* is dramatic and even puzzling (see Table 1). This gradual increase of *ν* is most likely due to the transition from Anderson MIT to BKKT (where, by definition, *ν* → ∞, that occurs whenever *B* > *B*_*c*_).

It is known that at strong magnetic fields the Pruisken-Khmelnitzkii renormalization-group (RG) formalism is based on the two parameters *σ*_*xx*_ and *σ*_*xy*_. Inclusion of SOS probably requires an additional parameter in the RG scheme, making it necessary to study the RG flow in three-dimensional space. Such an advanced calculation is beyond the scope of this work. However, we would like to analyze the results based on the general and standard RG approach^23^. Generically one has several fixed points, each with its own basin of attraction, separated by separatrices, such that crossing a separatrix corresponds to a phase transition. In this case, all the physical points within the same basin of attraction flow to the same fixed point, and are described by the same critical exponent. This is the case, for example, for the Anderson transition for finite SOS at zero magnetic field. In accordance with these lines that a RG flow persists for finite small magnetic fields until the separatrix to the BKT fixed points at *B*_*c*_, we want to have a single power-law divergence for *B* < *B*_*c*_ and a BKTT above. So we expect the following behavior,


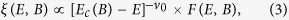


such that *F*(*E, B*) will be some number in the limit *E* → *E*_*c*_(*B*) for *B* < *B*_*c*_, and 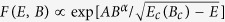 for *B* ≥ *B*_*c*_. A possible choice is


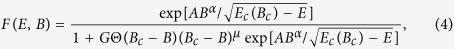


where *G* and *μ* are arbitrary constants. The effective crossover exponent *ν*(*B*) is defined by


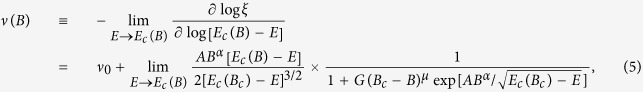


where we eliminate the step function since we are looking at *B* < *B*_*c*_. If we were to estimate *E*_*c*_(*B*) perfectly, then, as mentioned above, the critical exponent will remain *ν*_0_ all the way to *B*_*c*_. However, if we have an error because of the presence of the critical point at *B*_*c*_, then our estimation 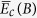 of *E*_*c*_(*B*) will be somewhere between the true *E*_*c*_(*B*) and *E*_*c*_(*B*_*c*_), say 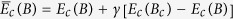. Such that we substitute 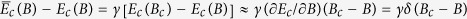 in the above expression:


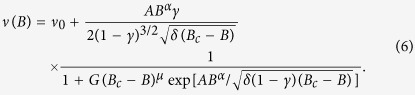


**Figure 3:**
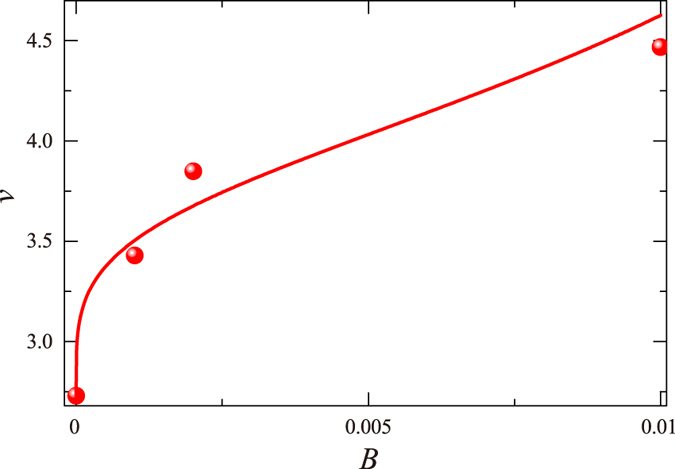
The effective crossover exponent *ν*(*B*) (red curve) shows a good agreement with the numerical data (red spots).

Fitting the numerical data with the expression above (see Fig. 3), we get 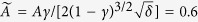, *α* = 1/4, and *B*_*c*_ = 1/50 (*G* = 0.02 but is immaterial). The effective crossover exponent *ν*(*B*) demonstrates a good agreement with the numerical data. It is also shown there that the critical magnetic field is 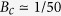. The two analyses confirm the existence of extended states for 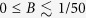. Thus, our results indicate that in the presence of both magnetic field and strongly fluctuating spin-orbit interaction, the one-parameter scaling theory of localization fails and one needs more than one length scale to characterize the system.

### Phase diagram

**Figure 4:**
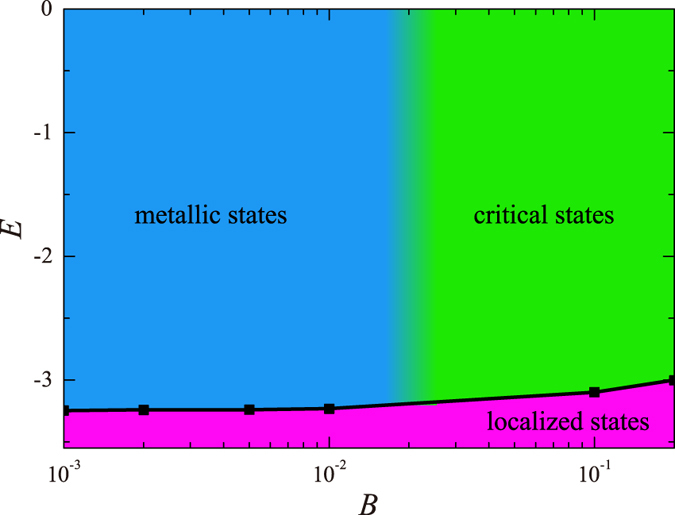
Schematic phase diagram in the *E*-*B* plane displaying the occurrence of three phases of localized states (pink), metallic states (blue), and critical states (green). Here the metallic states spread over the whole space (e.g. plane waves and Bloch states) which are distinct from the critical states who possess non-trivial multi-fractal structure. The black curve is *E*_*c*_(*B*). See text for further details.

A broader picture of the nature of states in the 2DSU model is obtained by combining the results of the present study with those of ref. 14, where the existence of a band of critical states at strong magnetic fields (*B* ≥ 1/5) has been demonstrated. It is found that *E*_*c*_(*B*) is a slowly increasing function, and that somewhere around 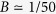 the Anderson MIT (discussed here) crosses over into a BKTT discussed previously^14^. Elucidating the nature of this crossover is beyond our scope. It requires the calculations of 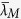 for many points in the *E*-*B* plane, with the hope to establish a critical curve separating the two bands of metallic and critical states. The resulting phase diagram in the *E*-*B* plane is depicted in Fig. 4. The emerging picture is that the band of extended states known to exist at *B* = 0, persists for finite *B*, until strong enough magnetic field 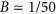 it crosses over (either sharply or smoothly) into a band of critical states as discussed in ref. 14.

## Discussion

Starting from the 2DSU model Hamiltonian (1), we focus on the localization issue at the weak field regime, starting at *B* = 0 where it is known to display MIT for system with the symplectic symmetry. Based on analyses of level statistics (Fig. 1) and localization length (Fig. 2), it has been demonstrated that a band of metallic states persists also for finite magnetic field 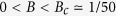. Combined with our previous results^14^, we can suggest a schematic phase diagram in Fig. 4, that elucidates the nature of localization in the *E*-*B* plane under the influence of spatially random spin-orbit potential. Thus, the paradigm that all states in 2D disordered systems with unitary symmetry are localized should be reviewed when strong spin-orbit fluctuations are present. In other words, in contrast to the prediction of the one-parameter scaling theory of localization^3^, localization in 2D disordered systems is not unambiguously determined by its symmetry. This suggests that, similar to what happens in the quantum Hall regime (occurring at strong magnetic field, without SOS), a second parameter is required to describe the scaling of the dimensionless conductance. Obvious questions are how to introduce such a parameter, and how the RG flow will look like in the presence of this additional parameter. Presently, the answers remain a theoretical challenge. To experimentally detect our results, we predict that in thin layers of Mott insulators with spin-orbit coupling (like 5*d* transition metal oxides SrIrO_3_ and Sr_2_IrO_4_^24^,^25^,^26^), the novel MIT occurs in the presence of small magnetic field.

Remarkably, (and unlike the localization issue), level statistics is found to be determined solely by symmetry, whether states are metallic or critical. As shown in Fig. 1, for *B* = 0, *P*(*s*) follows the Wigner surmise for the GSE, while for *B* = 1/10100, *P*(*s*) follows the Wigner surmise for the GUE. Moreover, *P*(*s*) obeys the GUE statistics also for the band of critical states discussed in ref. 14. This latter band is obtained following BKTT at strong magnetic field. In contrast, for critical states around a mobility edge in a standard Anderson MIT, a novel *P*(*s*) statistics is suggested^27^,^28^,^29^. What we conclude here is that *P*(*s*) is the same for metallic and critical states and depends solely on symmetry.

## Methods

In this section we show how to realize weak magnetic fields in a finite lattice model with periodic boundary conditions. Within the standard procedure of the Azbel-Hofstadter butterfly problem, one considers a square lattice of constant *a* and size *q* × *q* (where *q* is an integer) with site coordinates (*na, ma*) ≡ (*n, m*), and imposes the Landau gauge *A*_*x*_ = *By*. This means putting a vector potential equals *ϕ*_0_*mp*/(*qa*) on the link joining sites (*n, m*) and (*n* + 1, *m*), where *ϕ*_0_ = *hc*/*e* is the flux quantum and *p* = 1, 2, …, *q* represents the strength of the magnetic field. All site coordinates are considered modulo *q* to assure periodic boundary conditions. The magnetic flux per square is then equal to *ϕ*_0_*p*/*q* and the magnetic flux through the entire system is *ϕ*_0_*pq*.

In many cases, however, we need to tune the variation of flux through the entire system by a much smaller amount. As in the main text, we would like to study the system at very low magnetic fields, such that the total flux through the entire system is just *ϕ*_0_ and not *qϕ*_0_. Beside the important physical aspect, there is also a natural curiosity to expose how the energy curves behave “in between” the grid points *p*/*q* and (*p* + 1)/*q*. Here we suggest a very simple construction that requires a slight deviation of the geometry from a perfect square system, but this should not affect the physics in any way.

Consider a square lattice of size (*q* + 1) × *q* and vector potentials


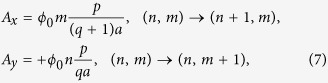


the +sign before *A*_*y*_ is in contrast with the symmetric gauge, namely (*A*_*x*_, *A*_*y*_) = ^→^↑. Since |*A*_*y*_| is just slightly greater than |*A*_*x*_| and they are counter-oriented, we call this construction an almost antisymmetric gauge. The total flux per square is then


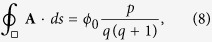


and the flux through the entire system is *pϕ*_0_ with *p* = 1, 2, …, *q*(*q* + 1). Thus, the minimum flux through the entire system is just by *ϕ*_0_.

The above construction can easily be checked for consistency when *p* divides *q*(*q* + 1). For example, when *p* = (*q* + 1) the flux per square is *ϕ*_0_/*q* and the spectrum can be obtained by solving the problem either with the gauge (7) with *p* = (*q* + 1) or with the Landau gauge *A*_*y*_ = *ϕ*_0_ × 1/*q*, following the substitution *ψ*_*nm*_ = 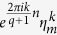, and solving the set of Harper equations with *k* = 1, 2, …*q* + 1, *m* = 1, 2, …*q* applied to the original rectangle of size (*q* + 1)*qa*^2^ (in this case the argument of the cosine function is 2*π*[*k*/(*q* + 1) + *mp*/*q*]) and *p* = 1, 2, …*q*. The corresponding spectra should then be identical. We have checked that this is indeed the case.

## Additional Information

**How to cite this article**: Su, Y. *et al*. Absence of localization in disordered two-dimensional electron gas at weak magnetic field and strong spin-orbit coupling. *Sci. Rep.* **6**, 33304; doi: 10.1038/srep33304 (2016).

## Figures and Tables

**Table 1:** Table of the critical energy *E*_*c*_, correlation length exponent *ν*, and reduced chi square 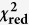 for different values of magnetic field.

*B*	*E*_*c*_	*ν*	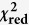
0	−3.259 ± 0.005	2.73 ± 0.02	0.927
1/1000	−3.245 ± 0.001	3.43 ± 0.08	0.843
1/500	−3.242 ± 0.002	3.85 ± 0.10	0.876
1/100	−3.232 ± 0.002	4.47 ± 0.15	0.890
